# An Isolated Enteric Duplication Cyst With Mucinous Neoplasm Presenting as a Mesenteric Cyst: A Case Report

**DOI:** 10.7759/cureus.72482

**Published:** 2024-10-27

**Authors:** Mert Uzunkulaoglu, Buse Nur Uge, Yiğit Düzköylü, Nevra Dursun Kepkep, Aytul Hande Yardimci

**Affiliations:** 1 General Surgery, Başakşehir Çam and Sakura City Hospital, Istanbul, TUR; 2 Pathology, Başakşehir Çam and Sakura City Hospital, Istanbul, TUR; 3 Gastroenterological Surgery, Başakşehir Çam and Sakura City Hospital, Istanbul, TUR; 4 Radiology, Başakşehir Çam and Sakura City Hospital, Istanbul, TUR

**Keywords:** case report, duplication cyst, infrequent intraabdominal lesions, malignant transformation, mucinous neoplasm

## Abstract

Enteric duplication cysts (EDCs) are infrequent intraabdominal lesions, though they can be detected anywhere throughout the gastrointestinal tract. Rarely, they can be asymptomatic and encountered in adulthood. Completely isolated EDCs are rarer with malignant transformation inside the cyst. Herein, we report a unique case of a mucinous neoplasm that developed in an isolated EDC in an adult patient treated with surgical resection and provide detailed radiological and histopathological findings.

## Introduction

Enteric duplication cysts (EDC) are rare congenital malformations that can be found in any region of the gastrointestinal tract and are encountered in one out of 4,000-5,000 live births [1]. Although most of the cases are detected in infancy and childhood due to manifestations such as abdominal pain, vomiting, bleeding, intussusception, or obstruction, they can rarely be diagnosed in adults [2,3]. Malignant transformation arising from the cyst is a very uncommon entity and has been reported only a few times in the literature [4,5].

Histopathologically, EDCs are characterized as tubular or round in shape and have a smooth muscle coat with an epithelial layer including ectopic cells of the digestive system with or without mucus production [6,7]. EDCs commonly have an anatomic connection with the gastrointestinal tract, usually on the mesenteric side, and obtain their blood supply from the bowel. They are most frequently found in jejunum and ileum with a prevalence of 47%, followed by the colon, esophagus, and stomach [8,9]. But occasionally they can be detected as isolated cysts with or without a separate pedicle having their own muscular wall and blood supply [2,10,11]. A complete-total isolated enteric duplication cyst (IEDC) is a very rare entity occurring with a prevalence of one in every 10,000 live births [12].

Herein, we report a case of an IEDC including mucinous neoplasm diagnosed as a mesenteric cyst initially in a 66-year-old female adult operated in a tertiary center. To the best of our knowledge, our case is one of the first published reports in English language literature so far [13]. Our work is in accordance with the Surgical CAse REport (SCARE) criteria [8,14].

## Case presentation

A 66-year-old female patient was admitted to our clinic with ongoing complaints of abdominal pain, weight loss (17 kg in the past five months), dyspepsia, and intermittent vomiting. The patient was a non-smoker Caucasian with a BMI of 23. Physical examination and medical and surgical history were unremarkable. Gastroscopy did not reveal any disorders other than mild antral gastritis without *Helicobacter pylori* positivity. Ultrasound examination was unremarkable.

Endoscopic ultrasound examination demonstrated a cystic mass with a diameter of 6 cm near the pancreas tail but without continuity with the pancreatic tissue. It was not suitable for fine needle aspiration because of the hindrance by small bowel segments. The common bile duct was dilated in the proximal segment, measured to be 13 mm in diameter, without any intraluminal pathology. The origin of the lesion could not be defined clearly.

Magnetic resonance imaging (MRI)

**Figure 1:**
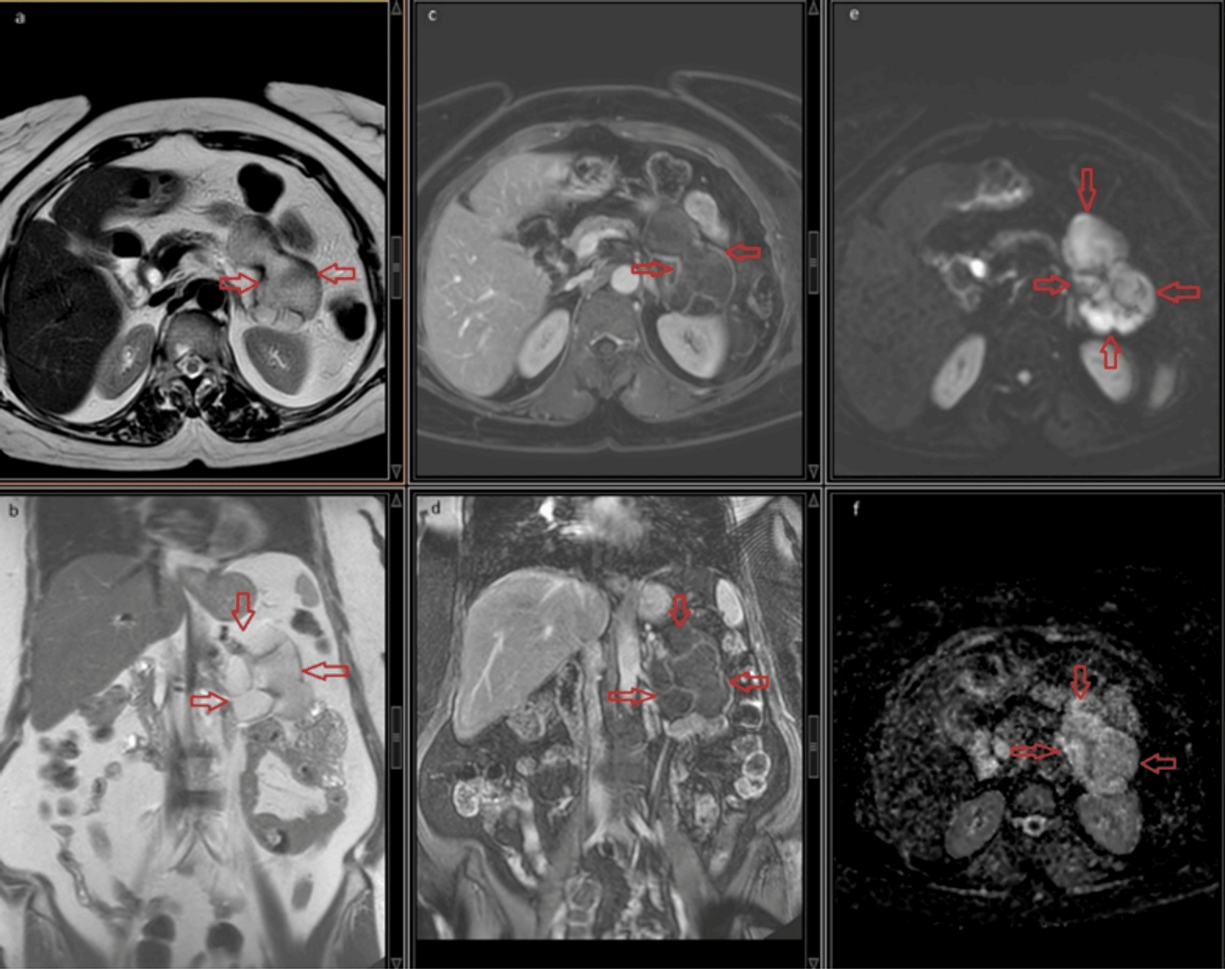
(a,b) Intracystic fluid as evidenced by high intensity on T2WI in the axial-coronal plane (arrows). (c,d) Significant multilobular formation and slight thickening of contrast enhancement along the septal wall (arrows). (e,f) Absence of any significant restriction or solid component (arrows). T2WI: T2-weighted imaging

Gadolinium-enhanced MRI clearly showed the intracystic fluid to be dense or mucinous, as evidenced by high intensity on T2-weighted imaging (T2WI) in the axial-coronal plane (Figures 1a-1b) and slightly high intensity on axial-coronal, fat-suppressed, contrast-enhanced T1-weighted imaging (T1WI) (Figures 1c-1d). The contrast-enhanced images clearly demonstrated significant multilobular formation and slight thickening of contrast enhancement along the septal wall. Notably, the diffusion-weighted MRI (Figures 1e-1f) did not reveal any significant restriction or solid component in any of the MRI sequences.

Surgery

Following the informed consent of the patient, open surgery was planned with the prediagnosis of a mesenteric cyst causing obstructive symptoms. In surgical exploration, there were no signs of malignant dissemination, free fluid, or invasion. The cystic lesion was located between the pancreatic tail, splenic hilus, and left renal capsule. It was found to be isolated without any anatomical attachment with any of those organs. The cystic wall was thickened without any macroscopic signs of malignant transformation. On first impression, the lesion was similar to a mesenteric cyst. The cyst was resected totally and was measured 10 cm in maximal diameter. Oral intake was started on the sixth hour of the postoperative period, and the patient was discharged from the hospital on the fourth day of the operation without any complications.

Gross and microscopy

**Figure 2:**
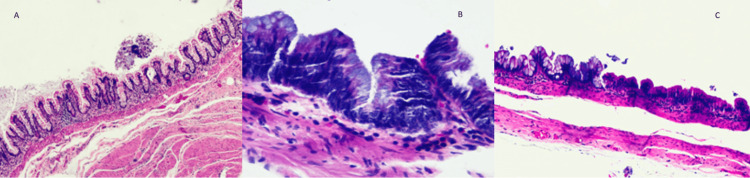
(A) Pseudostratified mucinous epithelium lined cyst wall without dysplasia, HEx10. (B) From low-grade dysplasia transition to high-grade dysplasia area, HEx20. (C) Pleomorphism and hyperchromatisa, with prominent nucleoli containing high-grade dysplasia mucinous epithelium, HEx40. HE: Hematoxylin and eosin

Macroscopic examination of the surgical specimen revealed a multilobulated cystic lesion measuring 10x9x5.5 cm. On sectioning, a thick, creamy, yellow material was found within the cyst. Microscopic examination revealed a pseudostratified mucinous epithelium with high-grade dysplasia (including increased distortion of gland architecture and crowded crypts) in some areas and low-grade dysplasia in others. There was an acellular mucin lake and hyalinization in the fibrotic background of the cyst wall, and no invasive focus was seen. The cyst was found to be positive for CK20, CDX2, and CK7 in immunohistochemical spacing. Figure 2 shows the enteric duplication cyst originating from the gastrointestinal system.

The patient was without any intraabdominal complaints or signs of recurrence at the sixth month postoperative follow-up. During follow-up, we performed a contrasted computed tomography (CT) scan in the third month and it did not reveal any pathology.

## Discussion

EDCs are infrequent intraabdominal lesions, and although they can be detected anywhere throughout the alimentary tract, jejunum and ileum are the most common when compared to other sites of the tract. They are usually localized on the mesenteric side of the intestines [15], with or without an anatomic tubular pedicle to the adjacent organ. They may also share the same wall and vascular system with the organ and have the same mucosal layer with that tract segment. Various theories have been suggested to explain the development of EDCs. One of them is the intrauterine vascular accident causing the occurrence of focal ischemic areas [16], while the other common theory is deteriorated luminal recanalization of the gastrointestinal tract [17]. Unfortunately, the exact underlying mechanism has not been explained yet.

Enteric cysts that are located in an isolated organ are rarer, reported as low as 1/10,000 to 1/100,000 live births [18]. They are usually presented in the neonatal period or mostly before the age of two years with male predominance [19]. They are extremely rare in adults, diagnosed incidentally as seen in our patient, and fewer than 50 patients have reported it so far [20,21]. Our case was of a 66-year-old female patient. Such kinds of cysts have their own blood supply, as was seen in our case. There are two main types of duplication cysts such as tubular and cystic types [22,23]. In our case, the lesion was cystic without any attachment to the neighboring organs.

Malignant transformation arising inside a cyst is a very rare entity and has only been reported a few times in the literature, including a retroperitoneal carcinoma and a poorly differentiated adenocarcinoma [24,25]. Here, we presented a case of an isolated EDC in an adult, localized between the pancreatic tail, spleen, and left kidney with mucinous neoplasm development, which is one of the first published cases in the literature [13].

The differential diagnosis for IEDCs around the pancreas may include benign or malignant pancreatic cystic lesions and mesenteric cysts [4]. In our case, our prediagnosis due to imaging was a mesenteric cystic.

There are no specific symptoms for IECDs, which leads clinicians to detect them incidentally, and the diagnosis is usually delayed as seen in our patient. Symptomatic cases of EDCs around the pancreas may cause recurrent symptoms such as pain and even pancreatitis [1]. Our patient had nausea, abdominal pain, and dyspepsia for over five years and was admitted to the family doctor a few times, receiving symptomatic treatment.

Ultrasound examination is usually ineffective in diagnosis [3,8] and fails to reveal the duplication, as was the case in our patient. Later, we performed an endoscopic ultrasound and detected a cystic lesion. Duplication cysts can be detected as smoothly surfaced, fluid-filled cystic lesions on cross-sectional imaging techniques [26]. Calcifications are rarely reported [27]. Radiological differential diagnoses include intraabdominal cystic lesions such as mesenteric and omental cysts, pancreatic pseudocysts, and even ovarian lesions [10]. In our patient, the MRI revealed intracystic fluid, significant multilobular formation, and slight thickening of contrast enhancement along the septal wall.

Although no clear standardized treatment method has been suggested for EDCs, surgical resection is accepted as the preferred method because of the presence of a symptomatic intraabdominal lesion [6,28] and the risk of malignancy even though it is very rare [29,30]. As in our case, incidental mucinous neoplasm was an unexpected result and surgery provided a curative treatment. We performed an en-bloc resection of the cystic lesion without any perioperative complications. We did not observe any noticeable attachments to the mesentery or pancreatic tail. The postoperative period was uneventful.

Histopathological examination revealed a pseudostratified mucinous epithelium with high-grade dysplasia in some areas and low-grade dysplasia in others, and it was positive for CK20, CDX2, CK 7 on immunohistochemistry, suggesting an enteric duplication cyst of gastrointestinal origin.

## Conclusions

We report a very rare case of mucinous neoplasm that developed in an isolated enteric duplication cyst in an adult patient. Preoperative imaging examination led us to a prediagnosis of a mesenteric cyst; however, histopathological diagnosis was consistent with this rare entity. The fact that EDCs can be encountered in adults with malignant transformation suggests surgical removal should be performed. The behavioral pattern of the case is difficult to estimate because of its rarity but we think that close follow-up is essential when similar cases of appendicial tumors are considered. A clinician should be cautious during follow-up because of the fact that such kinds of tumors are rare in the literature and their true malignancy potential is not clear.
